# BDNF Serum Levels are Associated With White Matter Microstructure in Schizophrenia - A Pilot Study

**DOI:** 10.3389/fpsyt.2020.00031

**Published:** 2020-02-21

**Authors:** Christine Hammans, Kristina Neugebauer, Vinod Kumar, Lea Mevissen, Melanie A. Sternkopf, Ana Novakovic, Tobias Wensing, Ute Habel, Ted Abel, Thomas Nickl-Jockschat

**Affiliations:** ^1^Department of Psychiatry, Psychotherapy and Psychosomatics, Faculty of Medicine, RWTH Aachen University, Aachen, Germany; ^2^JARA - Translational Brain Medicine, Jülich-Aachen Research Alliance, Jülich, Germany; ^3^Department of High-field Magnetic Resonance, Max-Planck-Institute for Biological Cybernetics, Tübingen, Germany; ^4^Institute of Neuroscience and Medicine: JARA-Institute Brain Structure Function Relationship (INM 10), Research Center Jülich, Jülich, Germany; ^5^Carver College of Medicine, Iowa Neuroscience Institute, University of Iowa, Iowa City, IA, United States; ^6^Department of Psychiatry, Carver College of Medicine, University of Iowa, Iowa City, IA, United States

**Keywords:** schizophrenia, brain derived neurotrophic factor, diffusion tensor imaging, Tract-based Spatial Statistics, neuroimaging, superior longitudinal fasciculus, dysconnectivity hypothesis

## Abstract

Brain derived neurotrophic factor (BDNF) has been implicated in the pathophysiology of schizophrenia. As BDNF regulates axonal and dendritic growth, altered BDNF levels in schizophrenia patients might underlie changes in structural connectivity that have been identified by magnetic resonance imaging (MRI). We investigated a possible correlation between BDNF serum levels, fiber tract architecture, and regional grey matter volumes in 19 schizophrenia patients and a gender- and age-matched control group. Two patients had to be excluded due to abnormalities in their MRI scans. Serum samples were obtained to determine BDNF levels, and T1- as well as diffusion-weighted sequences were acquired. We, then, investigated correlations between BDNF serum levels with neuroimaging parameters, using Voxel-based Morphometry (VBM) and Tract-based Spatial Statistics (TBSS). We found a significant negative correlation between BDNF serum levels and FA values in the right inferior fronto-occipital fasciculus and the right superior longitudinal fasciculus. These regions also showed a decrease in AD values in schizophrenia patients. Grey matter volumes were reduced in patients but there was no correlation between regional grey matter volumes and BDNF. The right superior longitudinal fasciculus has been repeatedly identified to exhibit microstructural changes in schizophrenia patients. Our findings of a negative correlation between BDNF and FA values in patients might indicate that BDNF is upregulated to compensate decreased structural connectivity as it induces neural plasticity and shows increased levels in damaged tissue. These findings of our pilot study are encouraging leads for future research in larger samples.

## Introduction

Schizophrenia is a severe chronic neuropsychiatric disorder (1–3) that shows a heritability of about 0.8 (4). More than 100 common variants associated with schizophrenia have been identified so far. These variants map to a large range of diverse genes. Given this diversity of the genes involved, a comprehensive understanding of common pathophysiological mechanisms is still lacking. However, recent studies have highlighted an association with synaptic plasticity for many of these susceptibility genes (5). Consequently, signaling pathways involved in the formation and maintenance of synaptic connections might constitute a common hub, over which all these diverse gene variants exert identical or related pathophysiological effects.

The neurotrophins are a protein family that might play a key role in this regard, as its members exert core functions in synapse formation, axonal and dendritic outgrowth, and other neuroplastic processes (6, 7). One of the members of the neurotrophin family, brain-derived growth factor (BDNF), is involved in the differentiation and growth of neurons (8). It maintains neurons and induces plasticity of neurons in the central nervous system, as well as in the peripheral nervous system (8–11). It has various functions in the brain, for example, the maintenance of cortical dendrites (12). BDNF is also involved in neuronal processes associated with learning and memory (8, 13). Mechanistically, BDNF influences neuronal plasticity by activating intracellular signaling cascades *via* TrkB receptors. These signalling cascades activate the transcription of genes that induce cell differentiation and survival of neurons (8), as well as axonal and dendritic branching and growth (8, 9). In addition, TrkB receptor-associated pathways affect glutamergic neurotransmission (11, 14), which is hypothesized to be hypofunctional in schizophrenia (15).

Altered serum levels of BDNF in patients compared to healthy volunteers were shown for many different neuropsychiatric diseases (e.g. Alzheimers, Epilepsy, Autism, Depression or Bipolar Disorder) (7, 16–19). In particular, meta-analyses showed a reduction of BDNF serum levels in schizophrenia with moderate effect sizes and heterogeneity (20, 21). Most original studies reported decreased BDNF levels (22–25) as well, while others, however, showed increases (26, 27) or no significant differences in BDNF serum levels (28). One potential explanation for these seemingly contradictory findings is the circadian rhythm of BDNF secretion (29). As not all studies determined BDNF serum levels at a standardized time of the day, this might have influenced the results. In addition, age, BMI, and duration of disease and medication might influence the results (21, 30) as well. Furthermore, there might be influencing factors not yet identified. Consequently, BDNF might mediate its effects *via* more intricate mechanisms than a mere reduction of overall levels.

These molecular mechanisms might—at least in part—underlie distinct alterations in brain structure in schizophrenia. Grey matter changes with mainly a fronto-temporo-thalamo-basal ganglionary pattern have been robustly reported across neuroimaging studies (31, 32). Improved imaging techniques, namely diffusion tensor imaging (DTI), in the last decade have also found changes in fiber tract architecture in schizophrenia patients. As schizophrenia has long been regarded as a disease of altered neural connectivity (33), DTI opened a possibility to study structural connectivity *in vivo*. Fiber tracts commonly reported to be altered are located in the frontal and temporal deep white matter, mainly within the dominant hemisphere (34–36) but also bihemispheric (37–39). In addition, long fiber tracts connecting regions of the frontal lobe, thalamus and cingulate gyrus as well as hippocampus and amygdala, and occipital lobe have been implicated to exhibit structural changes in schizophrenia (35, 36). These fiber tracts are identified to play a role in language and working memory, functions that are altered in schizophrenia (40, 41)

In this pilot study, we wanted to explore whether there was an association between micro- and mesostructural grey matter/fiber tract changes and BDNF serum levels in schizophrenia.

Although studies on a possible association of BDNF and grey matter have been previously published (42, 43), there are none comparing BDNF levels and white matter (fiber tracts) *in vivo*. Correlating BDNF levels with white matter fiber tracts and grey matter could provide initial clues if there was a relation between molecular parameters and the anatomical changes that are observed in patients.

Therefore, we a) obtained serum samples to determine BDNF concentrations; b) conducted MRI scans, including diffusion-weighed sequences, to analyze changes in grey matter and structural connectivity; and c) finally correlated BDNF serum levels with imaging parameters to gather further information about the impact of BDNF on the long fiber tracts.

## Materials and Methods

### Subjects

The study protocol was approved by the institutional review board of RWTH Aachen University Hospital, Aachen, Germany. A total of 20 healthy volunteers (12 male and 8 female) recruited from the local community and 19 patients with schizophrenia (11 male and 8 female) recruited at the Department of Psychiatry, Psychotherapy, and Psychosomatics, RWTH Aachen University Hospital, were enrolled in this study, as, in part, previously reported by Neugebauer and colleagues (44). Two of the patients had to be excluded due to abnormalities in their MRI data sets (one because of grossly enlarged lateral ventricles, most likely due to infantile hypoxia, and another one due to technical artifacts).Written informed consent was given by all participants prior to inclusion. The participants were matched for age, gender, and BMI as those criteria have been reported to exert an impact on BDNF levels as well as all participants were right handed due to the effects of handedness on neuroimaging parameters (30, 45–47). Inclusion criteria for patients and healthy controls were as follows: 1. Age between 18 and 55, 2. no history of a psychiatric disease for the healthy volunteers; for the patients exclusively, diagnosis of schizophrenia according to ICD-10 (F20.X) by an experienced clinician at RWTH Aachen University Hospital, 3. Right handedness, 4. German as mother language, and 5. Central European origin. Exclusion criteria were as follows for the patients and healthy controls: 1. general exclusion criteria for MRI, 2. known gross morphological cerebral abnormalities, 3. gravidity, 4. drug use, 5. infectious or chronical illness.

For all patients on treatment, the equivalent dose of olanzapine was estimated (**Table 1**) (48). The duration of disease was recorded for each patient, and schizophrenia symptoms were assessed with the Positive and Negative Syndrome Scale (PANSS).

**Table 1:** Detailed information on each patient's medication and duration of disease.

	Medication	Dosage	Olanzapine equivalent dose (48)	Duration of disease (in months)
Patient 1	Risperidone	4 mg	13,32	120
Patient 2	Amisulpride	200 mg	5,8	24
Patient 3	Citalopram	20 mg	20	21
Patient 4	Risperidone, Aripiprazole	6 mg, 15 mg	30	120
Patient 5	Amisulpride	300 mg	8,7	24
Patient 6	Quetiapine	1200 mg	32,4	336
Patient 7	Promethazine (on demand), Olanzapine	3x20 mg, 10 mg	10	192
Patient 8	Clozapine, Aripiprazole	450 mg, 25 mg	39,25	214
Patient 9	Quetiapine	450 mg	12,15	72
Patient 10	Movicol, Pantoprazole, Metformin, Sertindole, Prothipendyl (on demand)	1xd, 20 mg, 500 mg, 2 x 8 mg, 2 x 40 mg	16	144
Patient 11	Pregabalin, Amisulpride, Sertindole, Lorazepam (on demand)	200 mg, 1000 mg, 8 mg, 1 mg	8	144
Patient 12	Fluvoxamine, Clozapine; Amisulpride	25 mg, 425 mg, 600 mg	38,65	324
Patient 13	Clozapine, Fluvoxamine, Aripiprazole, Pantoprazole	150 mg, 50 mg, 10 mg,	14,2	396
Patient 14	Fluvoxamine, Clozapine, Gastrozepin, Paliperidone	25 mg, 250 mg, 50 mg, 250 mg/week	12,5 + 79,2857 = 91,7857	228
Patient 15	Amisulpride, Olanzapine	400 mg, 20 mg	31,6	180
Patient 16	Clozapine, Gastrozepin, Amisulpride, Azelastine	275 mg, 25 mg, 800 mg, 2x Hubs	13,75 + 23,2 = 36,95	36
Patient 17	Olanzapine; Citalopram	10 mg; 20 mg	10	12

Demographical and characteristic data as age, BMI, and gender was analyzed using SPSS 25 (SPSS, Inc., Chicago, IL, USA). We used t-tests to compare age and BMI and a X2 test to compare gender ratios between groups as seen in **Table 2**.

**Table 2:** Statistical analysis of the participants (HS, healthy subjects; SP, schizophrenia patients; N, number of subjects per group; BMI, Body Mass Index; BDNF, brain derived neurotrophic factor; ± Standard deviation; SE, standard error of the mean; PANSS - PANSS negative score, PANSS + - PANSS positive score; PANSS G - PANSS general score; ∑PANSS - PANSS total score).

	SP	HS
N	17	20
Gender (M/F)	10/7	12/8
Age in years	36.47 ± 10.02 SE 2.43	35.25 ± 11.51 SE 2.57
BMI	27.19 ± 5.14 SE 1.25	24.37 ± 4.71 SE 1.05
BDNF	15447.12 pg/ml ± 6967.21 SE 1557.92	16189.96 pg/ml ± 7177.76 SE 1740.86
Duration of disease in years	12.68 ± 9.92	
PANSS +	15.47 ± 6.34	
PANSS -	23.53 ± 9.84	
PANSS G	44.35 ± 16.1	
∑PANSS	24.65 ± 20.93	
Olanzapine Equivalent dose	24,65 ± 20,93	

### BDNF Serum Level Assessment

Blood samples were drawn from all participants at the same time (8 am) in the morning to account for circadian patterns of peripheral BDNF levels (29, 49). Two serum gel tubes were drawn from each participant and centrifuged at 2000 rpm for 10 minutes. The serum was pipetted into tubes and stored at –80°Celsius at the Department of Psychiatry, Psychotherapy, and Psychosomatics, RWTH Aachen University Hospital until further analysis. Standard enzyme linked immunosorbent assays (ELISAs) (Quantikine ELISA, Human BDNF Immunoassay, R&D Systems) (see **Supplementary Material**) were used to detect the actual serum concentration following the protocol provided by the manufacturer. All samples were analyzed in duplicate in one parallel session. To check for a normal distribution in the dataset, we used the Kolmogorov-Smirnov Test. As this test indeed indicated a normal distribution, we decided to use a two-sample t-test with an uncorrected p-value < 0.05 between BDNF and the two groups.

### MRI Data Acquisition

All MRI images were collected using a Siemens Trio 3T MRI scanner (Siemens Medical Systems, Erlangen, Germany) at the Department of Psychiatry, Psychotherapy, and Psychosomatics, RWTH Aachen University Hospital, immediately after the blood draw. A 32–channel coil was used to obtain the images. The head was immobilized using cushions to minimize head movement. In a session lasting 30 to 45 minutes, diffusion-weighted data, resting-state fMRI, and T1 anatomical sequences were acquired from all participants.

The T1 protocol for the Magnetization Prepared Rapid Acquisition Gradient Echo (MP-RAGE) sequence was as follows: sagittal slices 176, slice thickness = 1mm, field of view (FoV) = 250 mm, resolution matrix size 256 × 256 × 176, isotropic resolution of 1 mm, repetition time (TR)/echo time (TE)/inversion time (TI) = 1900 ms/2.52 ms/900 ms, flip angle (FP) = 9°, voxel size = 0.976 x 0.976 x 1 mm, duration = 7:49 min.

Diffusion-weighed sequences were acquired with 2.5 mm isotropic resolution, b-value of 1500 and 64 directions, and one non-diffusion image in each subject.

### MRI Analysis

The MRI images were inspected manually by an experienced clinician to exclude inadequate data sets, e.g., due to gross morphological structural abnormalities, technical and motion artefacts. We had to exclude two data sets of schizophrenia patients (one because of grossly enlarged lateral ventricles, most likely due to infantile hypoxia, and another due to technical artifacts). These two data sets were also excluded from the study regarding BDNF correlation.

### Voxel-Based Morphometry (VBM)

In brief, a VBM analysis was carried out as follows:

We used the Diffeomorphic Anatomical Registration Through Exponentiated Lie Algebra (DARTEL) segmentation algorithm of SPM 12 (50) for whole brain voxel-based morphometry (VBM) analysis on the NIFTI files of both schizophrenia patients and healthy controls. First, we conducted a group comparison between patients and controls. In a subsequent step, BDNF median values from two quantitative measurements were calculated and potential correlations with brain structural changes were investigated. All analyses were thresholded at a p-value of 0.05.

A detailed protocol of this approach can be found in Neugebauer et al. (44).

### Tract-Based Spatial Statistics (TBSS)

The analysis of the diffusion-weighed data sets was carried out using the standard FSL tool package (http://www.fmrib.ox.ac.uk/fsl/) for TBSS according to the standard protocol (51, 52). At first, all Siemens default output DICOM data sets were converted into NIFTI files with the dcm2nii tool, and the DICOM headers were used to determine the b-value and b-vector. “Then, voxel-wise statistical analysis of the Fractional Anisotropy (FA) data was carried out using TBSS [Tract-Based Spatial Statistics (51, 53), part of FSL (52, 53)]. First, FA images were created by fitting a tensor model to the raw diffusion data using FDT, and then brain-extraction using BET (54). All of the subjects' FA data were then aligned into a common space using the nonlinear registration tool, FNIRT (55, 56), which uses a b-spline representation of the registration warp field (57). Thereafter, the mean FA image was created and thinned to create a mean FA skeleton (we used a threshold for FA ≥ 0.2), which represents the centers of all tracts common to the group. Each subject's aligned FA data was then projected onto this skeleton and the resulting data fed into voxelwise cross-subject statistics”. We used a threshold for FA ≥ 0.2. Each step of this protocol was reviewed visually. We performed Voxel-wise group comparisons (healthy vs patients). BDNF regressions were performed for FA maps of both groups separately and one analysis for both groups combined using FSL randomise (58). The same protocol was carried out for Axial Diffusivity (AD), Mean Diffusivity (MD), and Radial Diffusivity (RD). All analyses were carried out using TFCE (Threshold-Free Cluster Enhancement) at a p-value < 0.05, corrected for family-wise error (FWE).

## Results

### BDNF Serum Levels

There was no significant difference between the BDNF serum levels of the healthy volunteers and the schizophrenia patients (p-value of 0.752). The mean BDNF level of the controls was 15447.12 pg/ml (SD 6967.21, SD mean 1557.92), and the mean BDNF level of the patients was 16189.96 pg/ml (SD 7177.76, SD mean 1740.86) (**Table 2**). There was one outliner in the patient group, but even with this outliner removed, differences between the two groups did not reach statistical significance.

### Regional Grey Matter Volumes and Their Correlations With BDNF Serum Levels

There were no significant correlations between regional grey matter volumes and BDNF serum levels.

As described previously, we found significant reductions of grey matter volume in schizophrenia patients in a widespread fronto-temporo-parietal network (44).

### White Matter Changes and Their Correlations With BDNF Serum Levels

**Figure 1:**
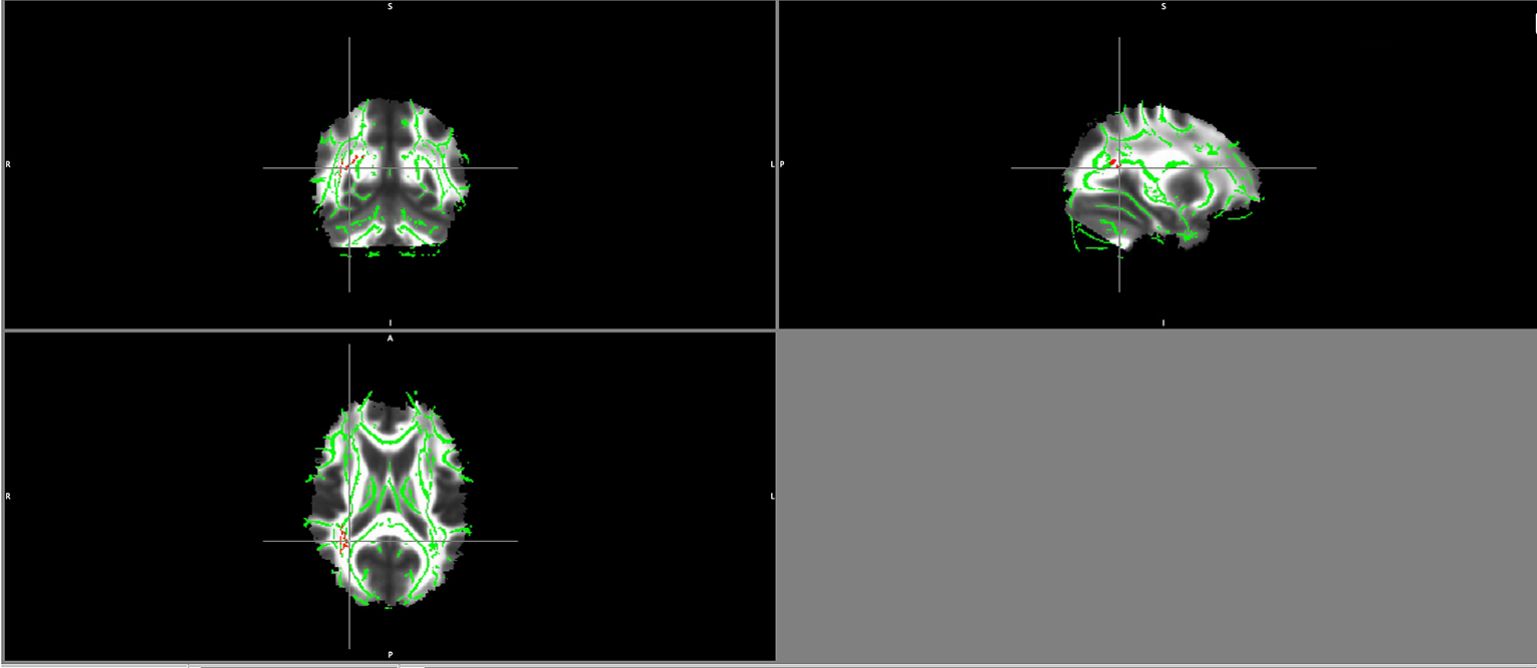
Lower AD values in patients maps in the right superior longitudinal fasciculus and right inferior fronto-occipital fasciculus (in red).

There were no significant differences between the healthy volunteers and schizophrenia patients regarding the group comparison of FA maps as well as MD and RD maps with a threshold of ≥ 0.2 and a p-value of 0.05 TFCE corrected. However, patients showed lower AD values in the right inferior fronto-occipital fasciculus and right superior longitudinal fasciculus, the same region where FA values correlated negatively with BDNF serum levels in patients (**Figure 1**).

**Figure 2:**
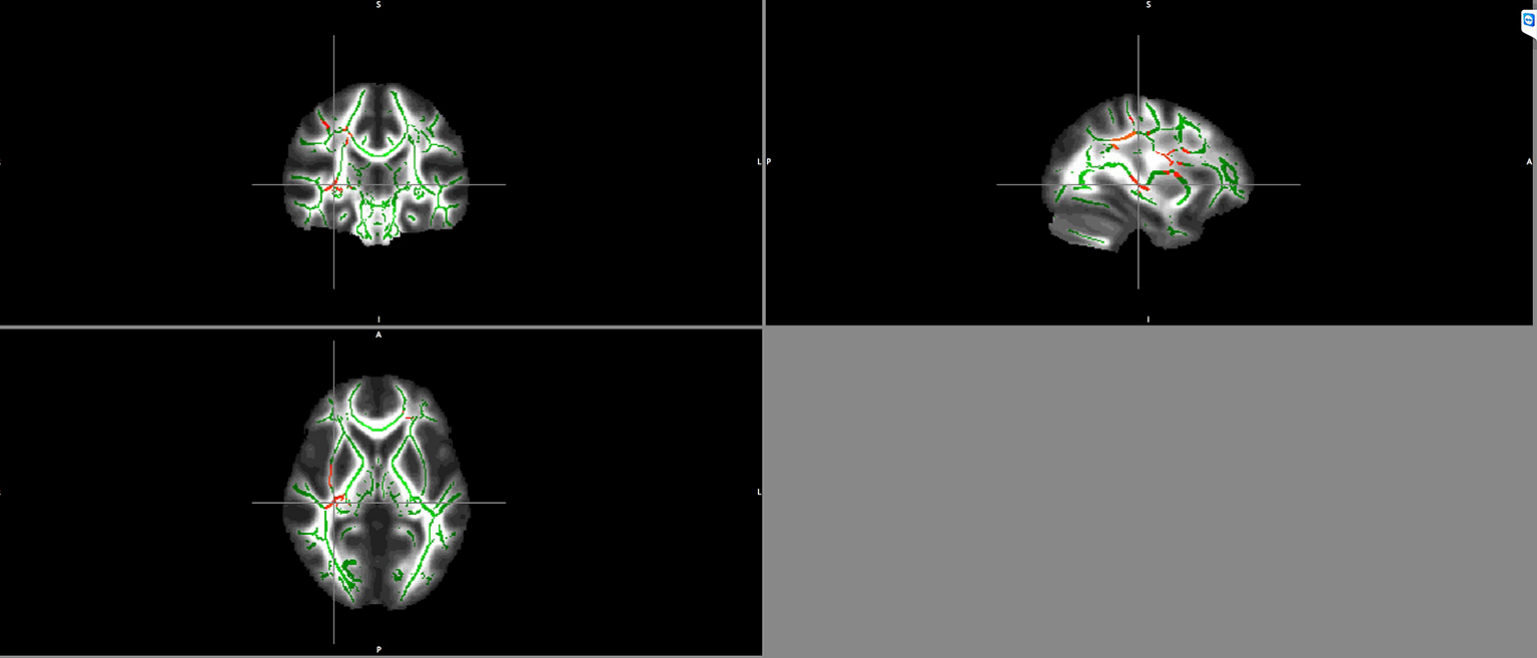
Negative correlation between BDNF serum levels and FA maps of patients in the right inferior fronto-occipital fasciculus and the right superior longitudinal fasciculus. Clusters identifying Voxels with significant negative correlations between FA and BDNF are highlighted in red.

Significant negative correlations between BDNF serum levels and FA values in schizophrenia patients were detected in the right inferior fronto-occipital fasciculus and the right superior longitudinal fasciculus (**Figure 2**).

## Discussion

Our pilot study was one of the first to explore potential correlations between BDNF serum levels and meso- and macroscopic imaging parameters in schizophrenia patients. Our findings could provide first hints at potential molecular mechanisms underlying these brain structural changes in patients.

### Neuroanatomical Changes in Patients Compared to Healthy Controls

We detected a decrease in AD in the right superior longitudinal fasciculus and right inferior fronto-occipital fasciculus in our patients, whereas RD did not change significantly. In general, AD is seen as a maker for axonal integrity (59, 60), whereas RD is seen as a marker for myelin integrity (61, 62). It is certainly challenging to infer from changes in DTI parameters on microstructural changes, especially in a small sample like ours. Nevertheless, it is tempting to speculate on the neurobiological underpinnings, as our study showed a decrease of AD in patients in the superior longitudinal fasciculus (SLF) and right inferior fronto-occipital fasciculus. We would interpret these reduced AD values as suggestive of a possible axonal pathology in schizophrenia patients. In particular, the SLF and the inferior fronto-occipital fasciculus are two areas that were previously shown to be altered in patients with schizophrenia (37, 63). Most studies found a decrease in FA values in these fiber tracts in schizophrenia patients (37, 63). However, only few studies (64–69) have investigated changes of AD and RD in schizophrenia so far. Their results were heterogenous. Some showed no difference in AD values compared to healthy controls, while others showed an increase of AD values accompanied by reduced FA values and increased RD values, which were interpreted as an indication for decreased myelinization of axons (70, 71).

In contrast to these prior studies, we found a decrease of AD values in schizophrenia patients. We interpreted it as possible alterations in axonal integrity. As schizophrenia is hypothesized to be a disease of impaired neural connectivity (72–75), changes in AD, hence, could indicate alterations in structural connectivity. Studies on the underlying histology (76, 77) showed that there is indeed decreased axonal tropism in brains of schizophrenia patients (78). In summary, we hypothesize that our results of reduced AD values in schizophrenia patients hint towards axonal changes as the “smallest common denominator” for schizophrenia patients.

The GMV in schizophrenia patients was reduced compared to healthy controls. These changes have repeatedly been interpreted as an indicator of perikaryal atrophy as a result of inactivity due to impaired synaptic plasticity (79, 80).

### A Potential Role for BDNF in Fiber Tract Changes of Schizophrenia Patients

Our finding of a negative correlation between BDNF serum levels and the FA values of patients in the right inferior fronto-occipital fasciculus and the superior longitudinal fasciculus (SLF) might seem contrary to the current understanding of BDNF values in schizophrenia. One might expect a positive correlation of BDNF and FA values, as BDNF is known to induce physiological differentiation and growth of neurons (8), especially in axonal and dendritic outgrowth as well as the maintenance of these cellular structures (6, 7, 12). The function of BDNF in a pathophysiological framework such as schizophrenia though should be met with caution, as for example, genome wide association studies in schizophrenia have shown variants in multiple genes regulating neuronal and synaptic plasticity and dendritic growth. As a result, it is commonly expected that these biological functions are mediated by dys-/hypofunctional signaling pathways in schizophrenia (5). Hence, seeing increased dendritic growth and synaptic plasticity as a result of upregulated BDNF in schizophrenia patients and therefore resulting in a positive correlation with FA seems unlikely. We would hypothesize that a negative correlation of BDNF and FA values in the SLF is an indicator for an ongoing response mechanism due to alterations in synaptic, dendritic, and axonal plasticity but that this response mechanism is not able to accomplish a physiological response as BDNF downstream signaling is impaired and therefore BDNF is incapable of inducing neuronal plasticity and further dendritic growth. Our own findings of a negative correlation between BDNF serum levels and FA values in the SLF of schizophrenia patients might reflect this dysfunctional neurotrophic mechanism. Due to its neurotrophic effects mentioned above, increased BDNF concentrations have been reported in brain regions with tissue damage, indicating a role for BDNF in neuronal repair mechanisms (9, 12, 81). Consequently, structural impairments of the SLF could lead to a compensatory increase of BDNF levels, however, potentially due to a disruption of neurotrophic downstream signaling cascades (5), this obviously does not lead to a restitution of this tissue. These findings would indicate that dysregulated BDNF levels in schizophrenia patients might point to impaired repair mechanisms with elevated serum concentrations as a response to altered structural connectivity, rather than an early step in the causal chain that leads to these alterations in connectivity. In addition, the SLF has been repeatedly indicated as altered in schizophrenia patients by various studies (63, 82); Studies found that FA values were decreased in the SLF (83–85) in patient populations. This was interpreted by the authors as a potential mesostructural correlation of altered neural plasticity.

Decreased AD values were found in the same region as the negative correlation between FA values and BDNF levels. We, thus, hypothesize that changes in AD are the smallest common dominator in schizophrenia patients, whereas our findings of a negative correlation of BDNF and FA values are a result of increased BDNF levels due to more global fiber tract changes, as FA is a summative parameter for microstructural integrity (e.g., axonal diameter and packing density, myelination, etc.) (86, 87).

**Figure 3:**
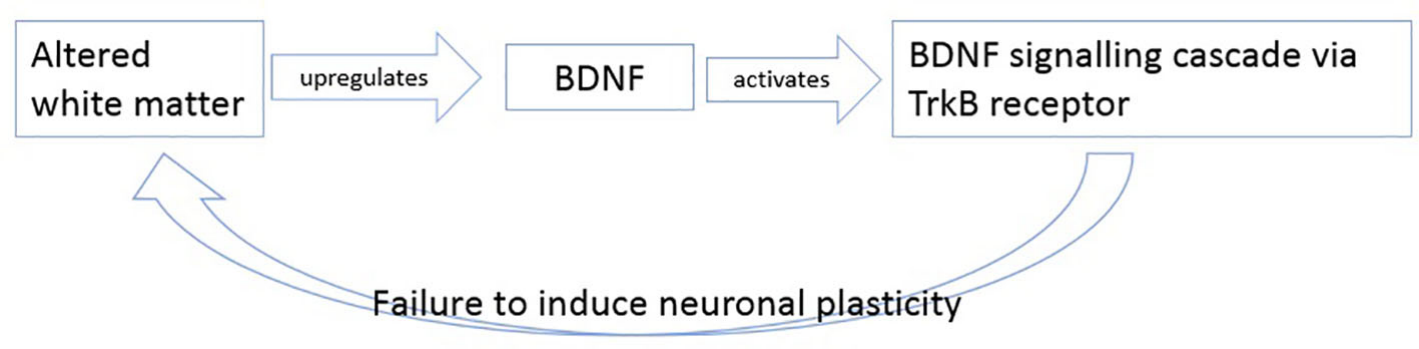
Description of the potential failure to induce neuronal plasticity in schizophrenia.

In summary, we hypothesize that BDNF levels increase as response to altered white matter possible axonal damage. The upregulated BDNF then tries to induce plasticity by activating its TrkB pathway, but as mentioned in literature, this signaling cascade is believed to be altered in patients with schizophrenia (26, 88, 89) so that BDNF cannot induce its effects of plasticity anymore (**Figure 3**).

There is also another important mechanism related to the pathophysiology of schizophrenia in which BDNF is involved in—glutamatergic neurotransmission. BDNF exerts its effects *via* two different pathways: TrkB and p57. Through TrkB activation, glutamate secretion and NMDA receptor function is modulated (14, 90). As stated in the disconnection hypothesis, modulation of synaptic plasticity through NMDA receptors is abnormal, so the growth of dendritic spines and axonal myelination is disrupted (33, 91). These dysfunctions might be associated with altered BDNF levels, as BDNF influences and—with higher levels—increases NMDA receptor-dependent myelination of axons (91). As we found a negative correlation between BDNF levels and FA maps in schizophrenia patients, it could be, as mentioned above, consecutive to altered structural connectivity in the SLF as a kind of feedback mechanism. The SLF is a central fiber tract connecting the frontal, temporal, occipital, and parietal lobe and has an impact in memory and spatial information processing (41), functions that are impaired in schizophrenia (84) and are also connected to BDNF. Through the TrkB receptor cascade, BDNF stimulates long time potentiation (LTP) in hippocampal neurona, which, in turn, is well known to play a key role in learning and memory (92). In addition, modification of glutamate levels influences characteristic positive and negative symptoms as well as disturbances in working memory (93).

### Comparison of AD, MD, and RD With BDNF Levels

AD, MD, and RD values did not significantly correlate with BDNF values of patients and healthy subjects. This can be interpreted in different ways. FA is a summative parameter for microstructural integrity (e.g., axonal diameter and packing density, myelination, etc.) (86, 87), in general, that is sensitive to a variety of microstructural changes. Neuroimaging findings, in turn, have been reported to exhibit higher variance in schizophrenia patients than in healthy control groups (36, 94). This heterogeneity within the patient group, in turn, might obscure changes in other DTI parameters, especially since the sample enrolled in our pilot study was comparatively small. It will be an important issue for future studies with larger samples to achieve a better characterization of the microstructural changes that correlated with BDNF serum levels.

### No Correlation of Grey Matter and BDNF Values

In addition, we did not find any correlation between BDNF and grey matter changes as suggested by earlier publications (42, 43). Our findings suggest that changes in BDNF serum levels in schizophrenia are mainly associated with pathologies in axons, dendrites, and/or myelination (9). Consequently, our findings would support a role for dysfunctional BDNF signaling as a failed repair mechanism in schizophrenia with consequences for neural connectivity (33, 95).

## Limitations

One major limitation of our study is certainly the comparatively small sample size. The small number also does not allow any sub-analyses that might yield important insights into the exact relationship between BDNF serum levels and brain structural changes. However, it deserves to be pointed out that our project was intended as a pilot study and is, to the best of our knowledge, the first to examine potential correlations between BDNF serum levels and white matter changes in schizophrenia. Consequently, little is known about a relationship between BDNF and white matter microstructure in actual schizophrenia patients. More studies with larger sample sizes are needed to corroborate our findings further.

Our study is based on a one-time assessment and did not realize a longitudinal approach. It might be that medication (20, 84) has an impact on brain volume and white matter integrity, as well as BDNF levels. Another contributing factor exerting an effect on brain anatomy, and most likely, BDNF levels, certainly is duration of the disease (96). There are a number of studies investigating a possible association of brain volume loss and antipsychotic medication (32, 97–99). One of the largest longitudinal datasets suggests a correlation between cumulative antipsychotic medication and progressive brain volume loss (97) as does a follow-up study on the same cohort (32). This has led to the idea of a causal relationship between antipsychotic medication and neuroanatomical changes in schizophrenia. However, other publications have discussed that notion critically (99). Studies regarding a correlation of white matter integrity and antipsychotic treatment Xiao et al. (100), Huang et al. (101) Cho et al. (102), Zeng et al. (84), McNabb et al. (66) described heterogenous results. Most report an effect of antipsychotic treatment on white matter, but have yielded different findings regarding the directionality of these changes (84, 100, 102). One study, for example, found improved white matter integrity after antipsychotic treatment (84), while another described an opposite association (102). Thus, it seems unclear at this point what effects antipsychotic treatment might have on white matter in schizophrenia patients. Nevertheless, we certainly cannot rule out a possible influence of antipsychotic medication on our findings. Also, the broad distribution in age (30, 103) and BMI in this small sample size might have influenced our results as well as there might be factors we are not yet aware of that might influence BDNF serum levels. It also remains opaque if the relationship between BDNF serum levels and white matter changes is present at the onset of schizophrenia or if such a relationship manifests throughout the course of the disease (104).

## Conclusion

To the best of our knowledge, this is the first study to investigate potential correlations between brain structural changes and BDNF serum levels in schizophrenia. We found a negative correlation between BDNF levels and FA values in the SLF, a fiber tract that connects frontal with temporal and also parietal and occipital regions and has been repeatedly implicated in schizophrenia. This negative correlation might reflect impaired repair mechanisms in schizophrenia patients. The lack of significant correlations between BDNF serum levels and grey matter changes highlights the importance of BDNF for synaptic plasticity, while it does not seem to have a significant pathophysiological effect for cell migration of perikaryal structure. Future studies enrolling larger collectives will have to corroborate these findings.

## Data Availability Statement

The datasets for this manuscript are not publicly available due to local IRB regulations. Requests to access the datasets should be directed to Christine Hammans, christine.hammans@rwth-aachen.de.

## Ethics Statement

The studies involving human participants were reviewed and approved by Ethics committee University Hospital Aachen. The patients/participants provided their written informed consent to participate in this study.

## Author Contributions

TN-J designed the study, supervised data acquisition, analysis, and interpretation and corrected the manuscript. LM, MS, and AN performed informed consent. CH and VK analyzed the MRI data. KN helped with data acquisition and analysis. CH wrote the manuscript, assisted with data acquisition, analyzed, and interpreted the data. TW, TA, and UH corrected the manuscript.

## Funding

This work was sponsored by the foundation program “START” of the Ministry of Science and Research of the state of North Rhine-Westphalia, Germany.

## Conflict of Interest

The authors declare that the research was conducted in the absence of any commercial or financial relationships that could be construed as a potential conflict of interest.
