# Supplementary material for: BDNF Serum Levels are Associated With White Matter Microstructure in Schizophrenia - A Pilot Study
Source: Front Psychiatry. 2020 Feb 21;11:31. doi: 10.3389/fpsyt.2020.00031 (PMC7046752; doi:10.3389/fpsyt.2020.00031)
Supplement: Supplementary file 1 [file DataSheet_1.pdf]

Quantikine® ELISA

Human Free BDNF Immunoassay

Catalog Number DBD00
SBD00
PDBD00

For the quantitative determination of human free Brain-Derived Neurotrophic Factor (BDNF) concentrations in cell culture supernates, serum, and platelet-poor plasma.

This package insert must be read in its entirety before using this product.
For research use only. Not for use in diagnostic procedures.

TABLE OF CONTENTS

SECTION	PAGE
INTRODUCTION	1
PRINCIPLE OF THE ASSAY	2
LIMITATIONS OF THE PROCEDURE	2
TECHNICAL HINTS	2
MATERIALS PROVIDED & STORAGE CONDITIONS	3
OTHER SUPPLIES REQUIRED	4
PRECAUTIONS	4
SAMPLE COLLECTION & STORAGE	4
SAMPLE PREPARATION	5
REAGENT PREPARATION	5
ASSAY PROCEDURE	6
CALCULATION OF RESULTS	7
TYPICAL DATA	7
PRECISION	8
RECOVERY	8
SENSITIVITY	8
LINEARITY	9
CALIBRATION	9
SAMPLE VALUES	10
SPECIFICITY	11
REFERENCES	12
PLATE LAYOUT	13

MANUFACTURED AND DISTRIBUTED BY:

USA & Canada | R&D Systems, Inc.
614 McKinley Place NE, Minneapolis, MN 55413, USA
TEL: (800) 343-7475 (612) 379-2956 FAX: (612) 656-4400
E-MAIL: info@RnDSystems.com

DISTRIBUTED BY:

UK & Europe | R&D Systems Europe, Ltd.
19 Barton Lane, Abingdon Science Park, Abingdon OX14 3NB, UK
TEL: +44 (0)1235 529449 FAX: +44 (0)1235 533420
E-MAIL: info@RnDSystems.co.uk

China | R&D Systems China Co., Ltd.
24A1 Hua Min Empire Plaza, 726 West Yan An Road, Shanghai PRC 200050
TEL: +86 (21) 52380373 FAX: +86 (21) 52371001
E-MAIL: info@RnDSystemsChina.com.cn

INTRODUCTION

Human mature BDNF is a 13 kDa, 119 amino acid (aa) residue non-glycosylated polypeptide whose primary structure is conserved among all mammalian species examined (1-4). Initially synthesized as a 247 aa residue prepropeptide, the BDNF molecule is divided into an 18 aa residue signal sequence, a 110 aa residue prosequence, and a 119 aa residue mature segment (2). Similar to other neurotrophic factors, there is a possibility that the N-terminus is alternatively spliced, giving rise to a longer pre-pro segment (but identical mature segment) with different functional properties (2). As a mature molecule, BDNF is 52% identical to NGF at the amino acid level, exists as a noncovalently-linked homodimer in solution, and contains six cysteine residues that are believed to form three intrachain disulfide linkages (1-3). Cells known to express BDNF include fibroblasts (5), astrocytes (6), neurons of varying phenotype and location (6-8), megakaryocytes/platelets (9), Schwann cells (near injury) (10) and, possibly, smooth muscle cells (11). BDNF in plasma is detected in the pg/mL range, while BDNF in serum is measured in the ng/mL range, the difference apparently attributable to platelet degranulation and BDNF release during clotting (3, 9, 12). The conservation of BDNF structure potentially allows a human BDNF ELISA to be widely applied across species.

There are at least two receptors for BDNF, the first being the low affinity 75 kDa Nerve Growth Factor Receptor (LNGFR), and the second being the high affinity 145 kDa TrkB (tropomyosin receptor kinase-B) (13). The LNGFR is a 399 aa residue Type-I (extracellular N-terminus) transmembrane glycoprotein that is currently considered to be a member of the TNF receptor superfamily (14, 15). Although all neurotrophins bind LNGFR with approximately the same affinity (Kd~1 nM), the significance of such binding is uncertain (13, 16, 17). What seems clear is that the LNGFR alone can engage certain signal transduction pathways (18). The biological significance of the activation of these pathways is not well understood. For BDNF specifically, LNGFR may serve as a retrograde transport molecule in neurons, promote Schwann cell migration near injury, and/or modulate TrkB activity in those cells that co-express both LNGFR and TrkB (16, 18). The second receptor for BDNF is the TrkB high affinity receptor (Kd~10 pM), a receptor that also binds NT-3 and NT-4/5 (13, 19). Human TrkB is a 792 aa residue type I (extracellular N-terminus) transmembrane glycoprotein that exhibits a number of distinct extracellular domains. These include two N-terminal cysteine-rich regions that flank an intervening leucine-rich domain and two membrane proximal C2 Ig-like domains (20). Comparing human and rat proteins, these regions exhibit over 90% identity in aa sequence (20). Alternative splice events have been identified for the TrkB gene in the mouse (21), the rat (22), and the human (20). In each case, nonsignalling, cytoplasmically-truncated variants are produced, leading to speculation that alternative splicing may be one method by which cells down-regulate neurotrophin activity (20, 22). While full TrkB activity is believed to require TrkB homodimerization (22), evidence suggests that full length TrkB and TrkC receptors may also form functional heterodimers in select cells where both receptors are co-expressed. These include cerebellar granule neurons and neurons of the hippocampal dentate nucleus (23, 24). Among the cells known to express TrkB are motoneurons of the spinal cord (10), pyramidal cells of the hippocampus (25), almost all neurons in the developing brain (25), and thymocytes (26), leading to speculation that BDNF plays a role in lymphopoiesis.

The number of functions attributed to BDNF is quite large. During development, BDNF has been implicated in neuronal differentiation, maturation, survival and synapse formation (27). In the adult, one of its most promising roles centers on neuroprotection, possibly protecting forebrain neurons from ischemic attack (28) and motor neurons from axotomy-induced death (29).

The Quantikine® Human Free BDNF Immunoassay is a 3.5 hour solid phase ELISA designed to measure free human BDNF in cell culture supernates, serum, and plasma not bound to other proteins, receptors, or other factors. It contains recombinant human BDNF expressed in *Sf* 21 cells and antibodies raised against the recombinant factor. This immunoassay has been shown to quantitate the recombinant BDNF accurately. Results obtained measuring natural human free BDNF showed dose-response curves that were parallel to the standard curves obtained using the Quantikine® kit standards. These results indicate that this kit can be used to determine relative mass values for natural human free BDNF.

PRINCIPLE OF THE ASSAY

This assay employs the quantitative sandwich enzyme immunoassay technique. A monoclonal antibody specific for human free BDNF has been pre-coated onto a microplate. Standards and samples are pipetted into the wells and any free BDNF present is bound by the immobilized antibody. An enzyme-linked monoclonal antibody specific for human free BDNF is added to the wells. Following a wash to remove any unbound antibody-enzyme reagent, a substrate solution is added to the wells and color develops in proportion to the amount of free BDNF bound in the initial step. The color development is stopped and the intensity of the color is measured.

LIMITATIONS OF THE PROCEDURE

- FOR RESEARCH USE ONLY. NOT FOR USE IN DIAGNOSTIC PROCEDURES.
- The kit should not be used beyond the expiration date on the kit label.
- Do not mix or substitute reagents with those from other lots or sources.
- It is important that the calibrator diluent selected for the standard curve be consistent with the samples being assayed.
- If samples generate values higher than the highest standard, dilute the samples with the appropriate calibrator diluent and repeat the assay. If cell culture supernate samples require large dilutions, perform an intermediate dilution with culture media and the final dilution with the appropriate calibrator diluent.
- Any variation in standard diluent, operator, pipetting technique, washing technique, incubation time or temperature, and kit age can cause variation in binding.
- Variations in sample collection, processing, and storage may cause sample value differences.
- This assay is designed to eliminate interference by other factors present in biological samples. Until all factors have been tested in the Quantikine® Immunoassay, the possibility of interference cannot be excluded.

TECHNICAL HINTS

- When mixing or reconstituting protein solutions, always avoid foaming.
- To avoid cross-contamination, change pipette tips between additions of each standard level, between sample additions, and between reagent additions. Also, use separate reservoirs for each reagent.
- To ensure accurate results, proper adhesion of plate sealers during incubation steps is necessary.
- When using an automated plate washer, adding a 30 second soak period following the addition of Wash Buffer, and/or rotating the plate 180 degrees between wash steps may improve assay precision.
- Substrate Solution should remain colorless until added to the plate. Keep Substrate Solution protected from light. Substrate Solution should change from colorless to gradations of blue.
- Stop Solution should be added to the plate in the same order as the Substrate Solution. The color developed in the wells will turn from blue to yellow upon addition of the Stop Solution. Wells that are green in color indicate that the Stop Solution has not mixed thoroughly with the Substrate Solution.

MATERIALS PROVIDED & STORAGE CONDITIONS

Store the unopened kit at 2-8 °C. Do not use past kit expiration date.

PART	PART #	CATALOG # DBD00	CATALOG # SBD00	DESCRIPTION	STORAGE OF OPENED/ RECONSTITUTED MATERIAL
**Human Free BDNF Microplate**	890388	1 plate	6 plates	96 well polystyrene microplate (12 strips of 8 wells) coated with a monoclonal antibody specific for human free BDNF.	Return unused wells to the foil pouch containing the desiccant pack. Reseal along entire edge of zip-seal. May be stored for up to 1 month at 2-8 °C.*
**Human Free BDNF Standard**	890390	2 vials	12 vials	Recombinant human BDNF in a buffered protein base with preservatives; lyophilized. *Refer to the vial label for reconstitution volume.*	Discard the BDNF stock solution after 4 hours. Use a fresh standard for each assay.
**Human Free BDNF Conjugate**	890389	1 vial	6 vials	11 mL/vial of a monoclonal antibody specific for human free BDNF conjugated to horseradish peroxidase with preservatives.	May be stored for up to 1 month at 2-8 °C.*
**Assay Diluent RD1S**	895137	1 vial	6 vials	11 mL/vial of a buffered protein base with preservatives.	
**Calibrator Diluent RD5K**	895119	1 vial	6 vials	21 mL/vial of a buffered protein base with preservatives. *For cell culture supernate samples.*	
**Calibrator Diluent RD6P**	895118	1 vial	6 vials	21 mL/vial of animal serum with preservatives. *For serum/plasma samples.*	
**Wash Buffer Concentrate**	895003	1 vial	6 vials	21 mL/vial of a 25-fold concentrated solution of buffered surfactant with preservative. *May turn yellow over time.*	
**Color Reagent A**	895000	1 vial	6 vials	12 mL/vial of stabilized hydrogen peroxide.	
**Color Reagent B**	895001	1 vial	6 vials	12 mL/vial of stabilized chromogen (tetramethylbenzidine).	
**Stop Solution**	895032	1 vial	6 vials	6 mL/vial of 2 N sulfuric acid.	
**Plate Sealers**	N/A	4 strips	24 strips	Adhesive strips.	

* Provided this is within the expiration date of the kit.

DBD00 contains sufficient materials to run an ELISA on one 96 well plate.
SBD00 (SixPak) contains sufficient materials to run ELISAs on six 96 well plates.

This kit is also available in a PharmPak (R&D Systems®, Catalog # PDBD00). PharmPaks contain sufficient materials to run ELISAs on 50 microplates. Specific vial counts of each component may vary. Refer to the literature accompanying your order for specific vial counts.

OTHER SUPPLIES REQUIRED

- Microplate reader capable of measuring absorbance at 450 nm, with the correction wavelength set at 540 nm or 570 nm.
- Pipettes and pipette tips.
- Deionized or distilled water.
- Squirt bottle, manifold dispenser, or automated microplate washer.
- Refrigerated centrifuge.
- 500 mL graduated cylinder.
- **Polypropylene** test tubes for dilution of standards and samples.
- Human Free BDNF Controls (optional; R&D Systems®, Catalog # QC22).

PRECAUTIONS

The Stop Solution provided with this kit is an acid solution.

Some components in this kit contain a preservative which may cause an allergic skin reaction. Avoid breathing mist.

Color Reagent B may cause skin, eye, and respiratory irritation. Avoid breathing fumes.

Wear protective gloves, clothing, eye, and face protection. Wash hands thoroughly after handling. Refer to the MSDS on our website prior to use.

SAMPLE COLLECTION & STORAGE

The sample collection and storage conditions listed below are intended as general guidelines. Sample stability has not been evaluated.

Cell Culture Supernates - Remove particulates by centrifugation and assay immediately or aliquot and store samples at ≤ -20 °C. Avoid repeated freeze-thaw cycles.

Serum - Use a serum separator tube (SST) and allow samples to clot for 30 minutes at room temperature before centrifugation for 15 minutes at 1000 x g. Remove serum and assay immediately or aliquot and store samples at ≤ -20 °C. Avoid repeated freeze-thaw cycles.

Platelet-poor Plasma - Collect plasma on ice using EDTA, heparin, or citrate as an anticoagulant. Centrifuge at 2-8 °C for 15 minutes at 1000 x g within 30 minutes of collection. An additional centrifugation step of the separated plasma at 10,000 x g for 10 minutes at 2-8 °C is recommended for complete platelet removal. Assay immediately or aliquot and store samples at ≤ -20 °C. Avoid repeated freeze-thaw cycles.

BDNF is present in platelet granules and is released upon platelet activation. Therefore, to measure circulating levels of BDNF, platelet-poor plasma should be collected for measurement. It should be noted that many protocols for plasma preparation, including procedures recommended by the Clinical Laboratory and Standards Institute (CLSI), result in incomplete removal of platelets from blood. This will cause variable and irreproducible results for assays of factors contained in platelets and released by platelet activation.

SAMPLE PREPARATION

Use polypropylene tubes.

Serum samples require at least a 20-fold dilution into Calibrator Diluent RD6P prior to the assay. A suggested 20-fold dilution is 10 µL of sample + 190 µL of Calibrator Diluent RD6P.

REAGENT PREPARATION

Bring all reagents to room temperature before use.

Wash Buffer - If crystals have formed in the concentrate, warm to room temperature and mix gently until the crystals have completely dissolved. Add 20 mL of Wash Buffer Concentrate to deionized or distilled water to prepare 500 mL of Wash Buffer.

Substrate Solution - Color Reagents A and B should be mixed together in equal volumes within 15 minutes of use. Protect from light. 200 µL of the resultant mixture is required per well.

Human Free BDNF Standard - **Refer to the vial label for reconstitution volume.** Reconstitute the Human Free BDNF Standard with Calibrator Diluent RD5K (*for cell culture supernate samples*) or Calibrator Diluent RD6P (*for serum/plasma samples*). This reconstitution produces a stock solution of 4,000 pg/mL. Allow the standard to sit for a minimum of 15 minutes with gentle agitation prior to making dilutions.

Use polypropylene tubes. Pipette 300 µL of Calibrator Diluent RD5K (*for cell culture supernate samples*) or Calibrator Diluent RD6P (*for serum/plasma samples*) into each tube. Use the stock solution to produce a dilution series (below). Mix each tube thoroughly before the next transfer. The undiluted Human Free BDNF Standard (4,000 pg/mL) serves as the high standard. The appropriate calibrator diluent serves as the zero standard (0 pg/mL).

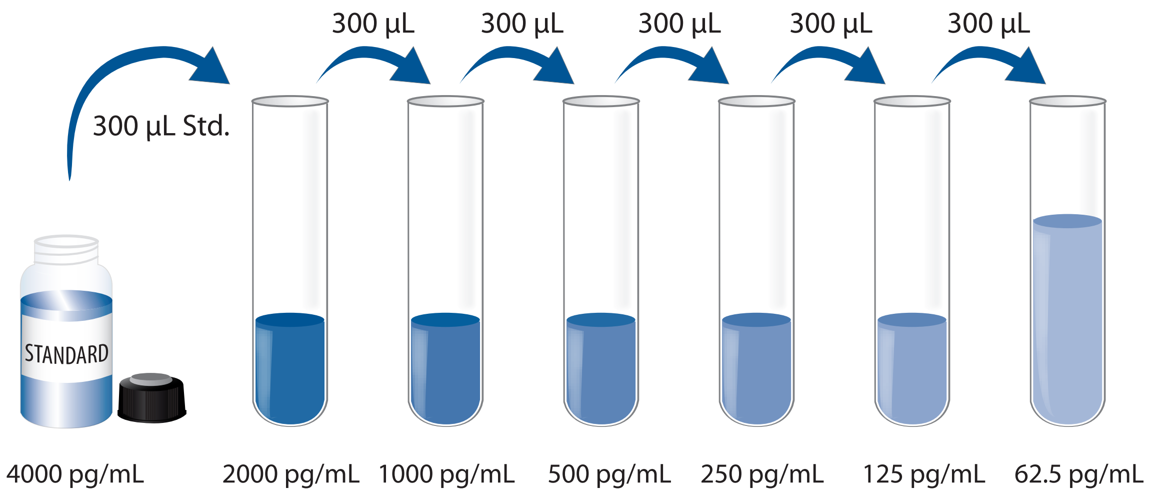

ASSAY PROCEDURE

Bring all reagents and samples to room temperature before use. It is recommended that all standards, samples, and controls be assayed in duplicate.

1. Prepare all reagents, working standards, and samples as directed in the previous sections.
2. Remove excess microplate strips from the plate frame, return them to the foil pouch containing the desiccant pack, and reseal.
3. Add 100 µL of Assay Diluent RD1S to each well.
4. Add 50 µL of standard, control, or sample* per well. Cover with the adhesive strip provided. Incubate for 2 hours at room temperature. A plate layout is provided to record standards and samples assayed.

   Note: ***Go to the next step. Do not wash.***
5. Add 100 µL of Human Free BDNF Conjugate to each well. Cover with a new adhesive strip. Incubate for 1 hour at room temperature.
6. Aspirate each well and wash, repeating the process twice for a total of three washes. Wash by filling each well with Wash Buffer (400 µL) using a squirt bottle, manifold dispenser, or autowasher. Complete removal of liquid at each step is essential to good performance. After the last wash, remove any remaining Wash Buffer by aspirating or decanting. Invert the plate and blot it against clean paper towels.
7. Add 200 µL of Substrate Solution to each well. Incubate for 30 minutes at room temperature. Protect from light.
8. Add 50 µL of Stop Solution to each well. The color in the wells should change from blue to yellow. If the color in the wells is green or the color change does not appear uniform, gently tap the plate to ensure thorough mixing.
9. Determine the optical density of each well within 30 minutes, using a microplate reader set to 450 nm. If wavelength correction is available, set to 540 nm or 570 nm. If wavelength correction is not available, subtract readings at 540 nm or 570 nm from the readings at 450 nm. This subtraction will correct for optical imperfections in the plate. Readings made directly at 450 nm without correction may be higher and less accurate.

*Samples may require dilution. See Sample Preparation section.

For research use only. Not for use in diagnostic procedures.

CALCULATION OF RESULTS

Average the duplicate readings for each standard, control, and sample and subtract the average zero standard optical density (O.D.).

Create a standard curve by reducing the data using computer software capable of generating a four parameter logistic (4-PL) curve-fit. As an alternative, construct a standard curve by plotting the mean absorbance for each standard on the y-axis against the concentration on the x-axis and draw a best fit curve through the points on the graph. The data may be linearized by plotting the log of the human free BDNF concentration versus the log of the O.D. and the best fit line can be determined by regression analysis. This procedure will produce an adequate but less precise fit of the data.

If samples have been diluted, the concentration read from the standard curve must be multiplied by the dilution factor.

TYPICAL DATA

These standard curves are provided for demonstration only. A standard curve should be generated for each set of samples assayed.

CELL CULTURE SUPERNATE ASSAY

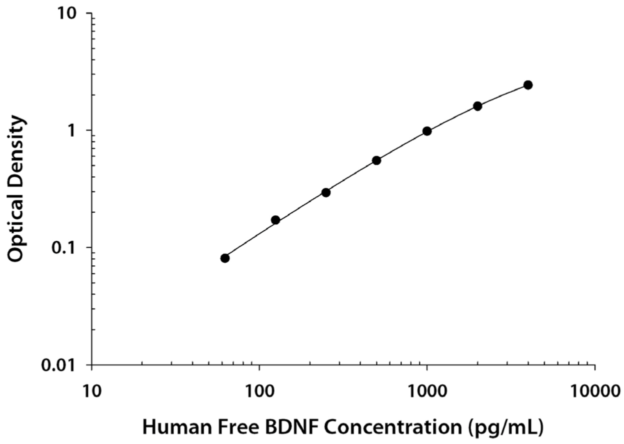

(pg/mL)	O.D.	Average	Corrected
0	0.033<br>0.034	0.034	—
62.5	0.114<br>0.116	0.115	0.081
125	0.188<br>0.225	0.206	0.172
250	0.323<br>0.332	0.328	0.294
500	0.578<br>0.594	0.586	0.552
1000	1.024<br>1.010	1.017	0.983
2000	1.624<br>1.647	1.636	1.602
4000	2.457<br>2.466	2.462	2.428

SERUM/PLASMA ASSAY

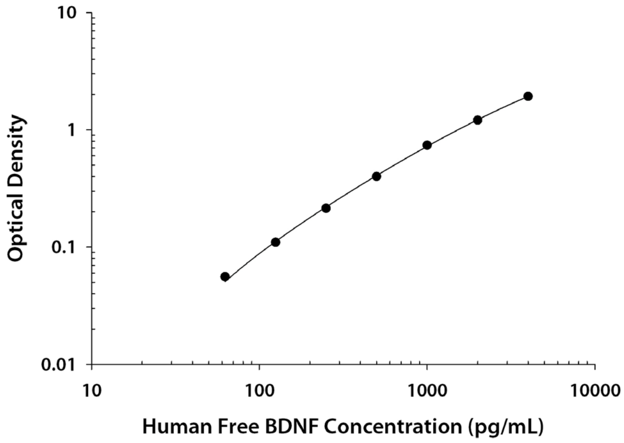

(pg/mL)	O.D.	Average	Corrected
0	0.030<br>0.030	0.030	—
62.5	0.087<br>0.084	0.086	0.056
125	0.140<br>0.140	0.140	0.110
250	0.238<br>0.249	0.244	0.214
500	0.434<br>0.425	0.430	0.400
1000	0.769<br>0.771	0.770	0.740
2000	1.242<br>1.233	1.238	1.208
4000	1.938<br>1.984	1.961	1.931

PRECISION

Intra-assay Precision (Precision within an assay)
Three samples of known concentration were tested twenty times on one plate to assess intra-assay precision.

Inter-assay Precision (Precision between assays)
Three samples of known concentration were tested in forty separate assays to assess inter-assay precision. Assays were performed by at least three technicians using two lots of components.

CELL CULTURE SUPERNATE ASSAY

	Intra-Assay Precision			Inter-Assay Precision		
**Sample**	**1**	**2**	**3**	**1**	**2**	**3**
**n**	20	20	20	40	40	40
**Mean (pg/mL)**	334	825	1339	358	905	1517
**Standard deviation**	15.6	28.3	56.5	36.8	86.8	121.6
**CV (%)**	4.7	3.4	4.2	10.3	9.6	8.1

SERUM/PLASMA ASSAY

	Intra-Assay Precision			Inter-Assay Precision		
**Sample**	**1**	**2**	**3**	**1**	**2**	**3**
**n**	20	20	20	40	40	40
**Mean (pg/mL)**	476	1258	2175	528	1338	2287
**Standard deviation**	24.0	48.2	135.7	59.7	102.3	186.0
**CV (%)**	5.0	3.8	6.2	11.3	7.6	8.1

RECOVERY

The recovery of human free BDNF spiked to three different levels throughout the range of the assay in various matrices was evaluated.

Sample Type	Average % Recovery	Range
Cell culture media (n=5)	96	83-105%
Serum* (n=5)	100	85-116%
Platelet-poor EDTA plasma (n=5)	96	89-115%
Platelet-poor heparin plasma (n=5)	93	81-119%
Platelet-poor citrate plasma (n=5)	99	87-120%

*Samples were diluted prior to assay.

SENSITIVITY

The minimum detectable dose (MDD) of human free BDNF is typically less than 20 pg/mL.

The MDD was determined by adding two standard deviations to the mean O.D. value of twenty zero standard replicates and calculating the corresponding concentration.

LINEARITY

To assess the linearity of the assay, samples containing and/or spiked with high concentrations of human free BDNF were diluted with calibrator diluent to produce samples with values within the dynamic range of the assay.

		Cell culture media (n=5)	Serum* (n=5)	Platelet-poor		
				EDTA plasma (n=5)	Heparin plasma (n=5)	Citrate plasma (n=5)
1:2	Average % of Expected	101	103	99	103	97
	Range (%)	100-102	97-110	92-105	95-109	89-105
1:4	Average % of Expected	101	104	99	105	97
	Range (%)	98-105	92-111	84-112	90-112	85-108
1:8	Average % of Expected	99	101	100	103	97
	Range (%)	94-101	90-110	85-112	87-110	83-106
1:16	Average % of Expected	100	104	105	108	101
	Range (%)	96-103	97-115	88-119	86-118	86-111

*Samples were diluted prior to assay.

CALIBRATION

This immunoassay is calibrated against a highly purified *Sf* 21-expressed recombinant human BDNF produced at R&D Systems®.

The NIBSC/WHO 1st WHO Reference Reagent 96/534 recombinant human BDNF Standard was evaluated in this assay. The dose response curve of the NIBSC Standard 96/534 parallels the Quantikine® standard curve. To convert sample values obtained with the Quantikine® Human Free BDNF kit to approximate NIBSC Units, use the equation below.

NIBSC (96/534) approximate value (IU/mL) = 0.0006 x Quantikine® Human Free BDNF value (pg/mL)

SAMPLE VALUES

Serum/Plasma - Samples from apparently healthy volunteers were evaluated for the presence of human free BDNF in this assay. No medical histories were available for the donors used in this study.

Sample Type	Mean of Detectable (pg/mL)	% Detectable	Range (pg/mL)
Serum* (n=33)	27,793	100	6186-42,580
Platelet-poor EDTA plasma (n=33)	428	56	ND-4137
Platelet-poor heparin plasma (n=33)	196	55	ND-528
Platelet-poor citrate plasma (n=33)	401	94	ND-1838

*Samples were diluted prior to assay.
ND=Non-detectable

Cell Culture Supernates - Human peripheral blood mononuclear cells (1 x 10^6 cells/mL) were cultured in RPMI supplemented with 10% fetal bovine serum, 50 µM β-mercaptoethanol, 2 mM L-glutamine, 100 U/mL penicillin, and 100 µg/mL streptomycin sulfate and stimulated with 10 µg/mL PHA. Aliquots of the cell culture supernates were removed on days 1 and 5 and assayed for levels of human free BDNF.

Condition	Day 1 (pg/mL)	Day 5 (pg/mL)
Unstimulated	132	109
Stimulated	160	104

SPECIFICITY

This assay recognizes natural and recombinant human free BDNF.

The factors listed below were prepared at 50 ng/mL in calibrator diluent and assayed for cross-reactivity. Preparations of the following factors prepared at 50 ng/mL in a mid-range recombinant human BDNF control were assayed for interference. No significant cross-reactivity or interference was observed.

Recombinant human:

CNTF
Pro-BDNF (aa19-128)
β-NGF
NGF R
NT-3
NT-4
TrkC

Recombinant mouse:

TrkC
TrkB
Pro-BDNF (aa19-130)
β-NGF
NGF R

Recombinant rat:

β-NGF

Recombinant human pro-BDNF (aa 19-247/R127A/R128A) cross-reacts approximately 13% in this assay.

Recombinant human TrkB did not cross-react in this assay, but demonstrated interference at all levels tested. TrkB was added at various concentrations to a mid-range BDNF control. The chart below lists the percentage that the control decreased out of the ± 3 SD range in the presence of the indicated concentrations of TrkB:

Human TrkB Concentration (ng/mL)	Percentage Decrease
50	87
25	79
12.5	64
6.3	47
3.1	26
1.6	7

REFERENCES

1. Leibrock, J. *et al.* (1989) Nature **341**:149.
2. Maisonpierre, P.C. *et al.* (1991) Genomics **10**:558.
3. Rosenfeld, R.D. *et al.* (1995) Protein Expr. Purif. **6**:465.
4. Barbacid, M. (1995) Curr. Opin. Cell Biol. **7**:148.
5. Cartwright, M. *et al.* (1994) Int. J. Dev. Neurosci. **12**:685.
6. Moretto, G. *et al.* (1994) J. Neuropathol. Exp. Neurol. **53**:78.
7. Barakat-Walter, I. (1996) J. Neurosci. Methods **68**:281.
8. Wetmore, C. *et al.* (1991) Proc. Natl. Acad. Sci. USA **88**:9843.
9. Yamamoto, H. and M.E. Gurney (1990) J. Neurosci. **10**:3469.
10. Griesbeck, O. *et al.* (1995) J. Neurosci. Res. **42**:21.
11. Scarisbrick, I.A. *et al.* (1993) J. Neurosci. **13**:875.
12. Radka, S.F. *et al.* (1996) Brain Res. **709**:122.
13. Eide, F.F. *et al.* (1993) Exp. Neurol. **121**:200.
14. Beutler, B. and C. van Huffel (1994) Science **264**:667.
15. Johnson, D. *et al.* (1986) Cell **47**:545.
16. Green, L.A. and D.R. Kaplan (1995) Curr. Opin. Neurobiol. **5**:579.
17. Wolf, D.E. *et al.* (1995) J. Biol. Chem. **270**:2133.
18. Carter, B.D. *et al.* (1996) Science **272**:542.
19. Dechant, G. *et al.* (1993) Development **119**:545.
20. Shelton, D.L. *et al.* (1995) J. Neurosci. **15**:477.
21. Klein, R. *et al.* (1990) Cell **61**:647.
22. Eide, F.F. *et al.* (1996) J. Neurosci. **16**:3123.
23. Minichiello, L. and R. Klein (1996) Genes and Dev. **10**:2849.
24. Canossa, M. *et al.* (1996) J. Biol. Chem. **271**:5812.
25. Barbacid, M. (1994) J. Neurobiol. **25**:1386.
26. Maroder, M. *et al.* (1996) J. Immunol. **157**:2864.
27. Henderson, C.E. (1996) Curr. Opin. Neurobiol. **6**:64.
28. Kokaia, Z. *et al.* (1996) Mol. Brain Res. **38**:139.
29. Kishino, A. *et al.* (1997) Exp. Neurol. **144**:273.

For research use only. Not for use in diagnostic procedures.

PLATE LAYOUT

Use this plate layout to record standards and samples assayed.

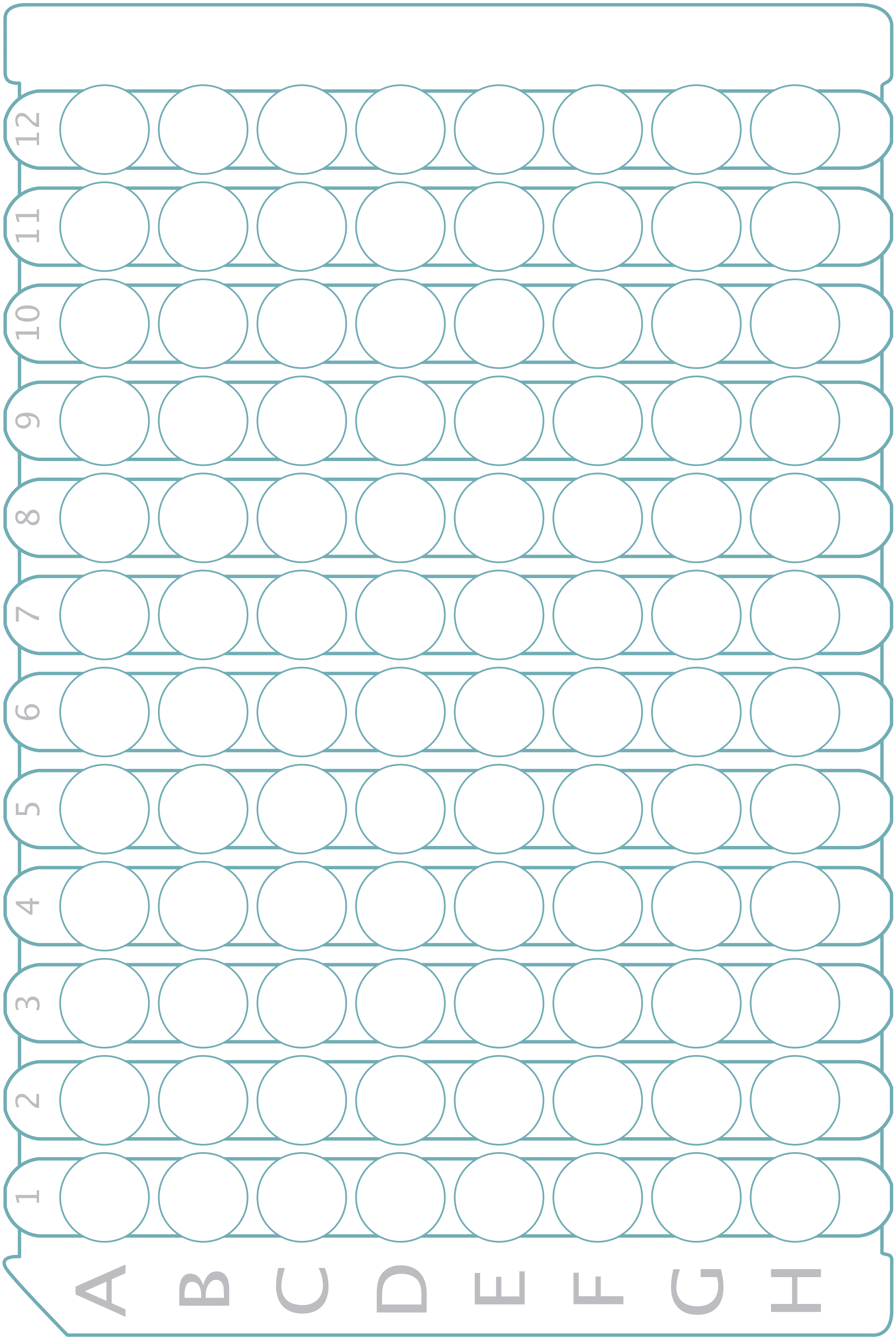

NOTES

All trademarks and registered trademarks are the property of their respective owners.

©2017 R&D Systems®, Inc.
